# Outcomes of renal replacement therapy in boys with prune belly syndrome: findings from the ESPN/ERA-EDTA Registry

**DOI:** 10.1007/s00467-017-3770-9

**Published:** 2017-08-04

**Authors:** Fatos Yalcinkaya, Marjolein Bonthuis, Beyza Doganay Erdogan, Karlijn J. van Stralen, Sergey Baiko, Hassib Chehade, Heather Maxwell, Giovanni Montini, Kai Rönnholm, Søren Schwartz Sørensen, Tim Ulinski, Enrico Verrina, Stefanie Weber, Jérôme Harambat, Franz Schaefer, Kitty J. Jager, Jaap W. Groothoff

**Affiliations:** 1 Department of Pediatric Nephrology, Ankara University Faculty of Medicine, Ankara, Turkey; 2 ESPN/ERA-EDTA Registry, Department of Medical Informatics, Academic Medical Center, University of Amsterdam, Amsterdam Public Health research institute, Amsterdam, The Netherlands; 3 Department of Biostatistics, Ankara University School of Medicine, Ankara, Turkey; 4 Spaarne Gasthuis Academie, Spaarne Gasthuis, Hoofddorp, The Netherlands; 5 Department of Pediatrics, Belarusian State Medical University, Minsk, Belarus; 6 Department of Pediatrics, Division of Pediatric Nephrology, University Hospital, Lausanne, Switzerland; 7 Department of Paediatric Nephrology, Royal Hospital for Sick Children, Glasgow, UK; 8 Pediatric Nephrology and Dialysis Unit, Department of Clinical Sciences and Community Health, University of Milan Fondazione IRCCS Cà Granda, Ospedale Maggiore Policlinico Via della Commenda, 9, 20122 Milan, Italy; 9 Children’s Hospital University of Helsinki, Helsinki, Finland; 10 Department of Nephrology, Rigshospitalet, Copenhagen University Hospital, Copenhagen, Denmark; 11 Department of Paediatric Nephrology, Armand-Trousseau Hospital, APHP, Paris, France; 12 University Pierre and Marie Curie, Paris, France; 13 Dialyisis Unit, Paediatric Nephrology and Dialysis Department, IRCCS Giannina Gaslini, Genoa, Italy; 14 University Children’s Hospital Marburg, Philipps-University, Marburg, Germany; 15 Pediatric Nephrology Unit, Bordeaux University Hospital, Bordeaux, France; 16 Department of Pediatric Nephrology, University Children’s Hospital Heidelberg, Heidelberg, Germany; 17 Department of Pediatric Nephrology, Emma Children’s Hospital, Academic Medical Center, Amsterdam, The Netherlands

**Keywords:** Prune belly syndrome, Children, Renal replacement therapy, Transplantation, Dialysis

## Abstract

**Background:**

As outcome data for prune belly syndrome (PBS) complicated by end-stage renal disease are scarce, we analyzed characteristics and outcomes of children with PBS using the European Society for Pediatric Nephrology/European Renal Association-European Dialysis and Transplant Association (ESPN/ERA-EDTA) Registry data.

**Methods:**

Data were available for 88 male PBS patients aged <20 years who started renal replacement therapy (RRT) between 1990 and 2013 in 35 European countries. Patient characteristics, survival, and transplantation outcomes were compared with those of male patients requiring RRT due to congenital obstructive uropathy (COU) and renal hypoplasia or dysplasia (RHD).

**Results:**

Median age at onset of RRT in PBS was lower [7.0; interquartile range (IQR) 0.9–12.2 years] than in COU (9.6; IQR: 3.0–14.1 years) and RHD (9.4; IQR: 2.7–14.2 years). Unadjusted 10-year patient survival was 85% for PBS, 94% for COU, and 91% for RHD. After adjustment for country, period, and age, PBS mortality was similar to that of RHD but higher compared with COU [hazard ratio (HR) 1.96, 95% confidence interval (CI) 1.03–3.74]. Seventy-four PBS patients (84%) received a first kidney transplant after a median time on dialysis of 8.4 (IQR 0.0–21.1) months. Outcomes with respect to time on dialysis before transplantation, chance of receiving a first transplant within 2 years after commencing RRT, and death-censored, adjusted risk of graft loss were similar for all groups.

**Conclusions:**

This study in the largest cohort of male patients with PBS receiving RRT to date demonstrates that outcomes are comparable with other congenital anomalies of the kidney and urinary tract, except for a slightly higher mortality risk compared with patients with COU.

## Introduction

Prune belly syndrome (PBS) is a very rare congenital disorder that consists of a deficiency in the development of anterior abdominal wall muscles, variable amounts of urinary tract dilatation, and cryptorchidism. Other fetal malformations that may be associated with PBS include gastrointestinal, cardiac, pulmonary, and limb abnormalities [1]. Prenatal ultrasound features might include oligo- or anhydramnios, megacystis, hydronephrosis, and hyperechogenic kidneys [2]. The incidence is 3.8/100,000 live births, and >95% of patients are boys [3]. Reports of more than one PBS case in the same family have suggested a genetic contribution. Although several gene loci have been defined, a clear genetic basis for PBS has not yet been established [4–7]. It is thought that PBS arises from a defect in the intermediate and lateral plate mesoderm development during the 6th to 10th week of gestation, resulting in clinical abnormalities [2, 8].

PBS is a clinical entity with a wide spectrum of severity; approximately 10–25% of newborn infants die in the perinatal period, and nearly a third of PBS patients outside the postnatal period will develop end-stage renal disease (ESRD) requiring renal replacement therapy (RRT) [9, 10]. Causes of ESRD are congenital renal dysplasia, lower urinary tract obstruction, and/or infection. These are similar to causes of ESRD seen in other congenital anomalies of the kidney and urinary tract (CAKUT), yet PBS has generally been associated with adverse outcomes in children on RRT [1]. However, reliable data on outcomes in these patients are lacking.

We aimed to describe the incidence, clinical, and demographic characteristics of boys with PBS requiring RRT using the European Society of Pediatric Nephrology/European Renal Association–European Dialysis and Transplant Association (ESPN/ERA–EDTA) Registry. In addition, long-term outcomes of PBS patients who started RRT between 1990 and 2013 were assessed and compared with male controls undergoing RRT for congenital obstructive uropathy (COU) and congenital renal hypodysplasia and dysplasia (RHD).

## Methods

### Study population

As PBS almost exclusively affects boys, girls were excluded from analyses. Patient data were extracted from the ESPN/ERA-EDTA Registry, a population-based cohort study. On an annual basis, the Registry collects individual patient data on all European children starting RRT, including date of birth, sex, primary renal disease, date and treatment modality at start of RRT, all subsequent changes in treatment modalities, and date and cause of death. Furthermore, data for several clinical parameters such as height and serum creatinine are collected for some countries [11].

The ERA-EDTA coding system for renal diseases was used to identify male patients with PBS [12] from 35 countries who started RRT at <20 years of age between January 1990 and December 2013. Two different groups of patients with other congenital causes of renal disease who commenced RRT in the same period served as controls: C(1) male patients with COU with or without vesicoureteric reflux and (2) those with congenital RHD. Table 1 describes participating countries and respective periods of follow-up. Additionally, we performed a sensitivity analysis comparing outcomes of PBS patients with those of glomerulonephritis patients on RRT. This sensitivity analysis did not yield any statistically significant differences in patient survival, access to transplantation, and graft survival between PBS and glomerulonephritis patients.

**Table 1:** Number of patients with prune belly syndrome and controls by country

Country (period)	Prune belly syndrome	Congenital obstructive uropathy	Renal hypo- or dysplasia
Austria (1990–2013)	2	22	30
Belarus (2008–2013)	2	7	5
Belgium (1993–2013)	1	0	9
Croatia (1990–2013)	1	14	3
Denmark (1990–2013)	1	24	15
Finland (1990–2013)	3	28	12
France (2004–2013)	6	72	156
Greece (1990–2013)	1	23	52
Italy (1994–2013)	9	1	0
Poland (2006–2013)	1	36	36
Portugal (2006–2013)	2	15	14
Spain (1990–2013)	13	119	43
Sweden (1990–2013)	4	0	58
Switzerland (1990–2013)	2	4	55
The Netherlands (1990–2013)	6	61	103
Turkey (2010–2013)	2	5	5
United Kingdom (1990–2013)	32	329	353
Other countries^1^	0	144	179
Total	88	904	1128

^1^Albania (2010–2013), Bulgaria (2008–2013), Czech Republic (1990–2013), Estonia (2007–2013), FYR of Macedonia (2006–2013), Georgia (2013), Germany (2010–2013), Hungary (2006–2013), Iceland (1990–2013), Lithuania (2006–2013), Montenegro (2007–2009), Norway (1990–2013), Romania (2006–2013), Russia (2006–2013), Serbia (2006–2013), Slovakia (2007–2013), Slovenia (2006–2013) and Ukraine (2010–2012)

Height values were normalized to Z-scores for age and sex using recent national growth charts or growth charts for northern or southern European countries [13]. Estimated glomerular filtration rate (eGFR) was calculated using the bedside Schwarz formula [14].

### Statistical analysis

To ensure complete coverage, incidence and prevalence of RRT for PBS were calculated for children <15 years of age and expressed per 100 million of the age-related population. General population data were derived from the Statistical Office of the European Communities (EUROSTAT) [15]. Only countries contributing data over the entire follow-up period (1990–2013) were included when calculating mean yearly incidence, as was the case for analyses of comparisons over time. Differences in patient characteristics between groups were examined using chi-square tests for categorical and Kruskal–Wallis test for continuous parameters. Patient and peritoneal dialysis (PD) technique survival rates were determined using the Kaplan–Meier method (unadjusted survival) and Cox proportional hazards frailty analyses (adjusted survival), including country as a random effect to account for clustering of patients within the same country. Causes of death were coded according to the ERA-EDTA coding system [12]. PD technique survival was defined as technique failure or switch to hemodialysis (HD), and patients were censored at the time of transplantation or death. Death was considered a competing event in access to transplantation and graft survival analyses, and a cumulative incidence competing risk method was used [16]. For patients with a pre-emptive kidney transplant, the time between start of RRT and transplantation was set to 0. Patients were followed until they were lost to follow-up, when reaching 20 years of age, or at the end of study (31 December 2013), whichever occurred first. Analyses were adjusted for country and age at onset and period of RRT. All statistical analyses were performed in SAS version 9.4 (SAS Institute Inc., Cary, NC, USA).

## Results

### Incidence and prevalence

Data were obtained on 2120 male patients. Of these, 88 (4.2%) had PBS, while 904 (42.6%) patients with COU and 1128 (53.2%) with RHD served as controls. PBS patients were identified in 17 countries (Table 1). Within countries with complete follow-up, the average yearly incidence of RRT for PBS in boys <15 years was 15.6 per 100 million age-related population in 1990–2013; point prevalence on 31 December 2013 of RRT for those boys was 68 per 100 million.

### Patient characteristics

**Fig. 1:**
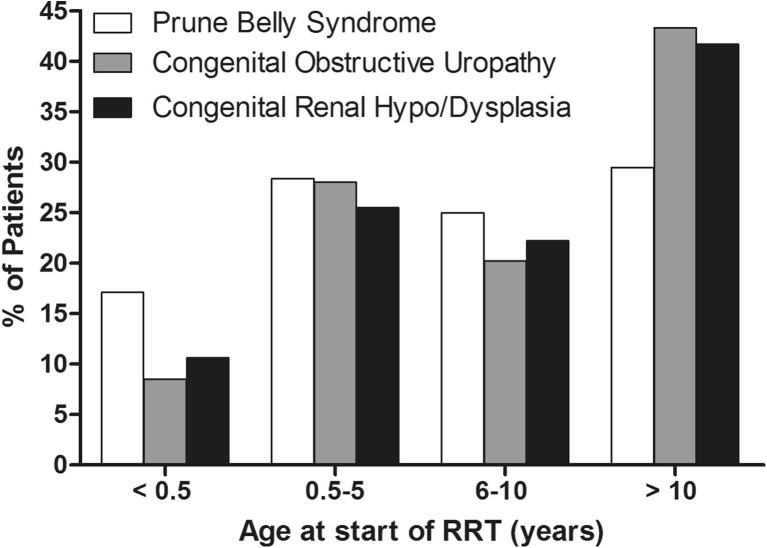
Age distributions of patients at the onset of renal replacement therapy (RRT)

Median age at onset of RRT was 7.0 [interquartile range (IQR) 0.9–12.2] years in PBS, i.e. significantly lower compared with patients with COU (9.6; IQR: 3.0–14.1 years) and RHD (9.4; IQR: 2.7–14.2 years) (*p* = 0.02). In age groups <6 months and between 6 and 10 years of age, a higher percentage of patients started RRT due to PBS than to COU or RHD (Fig. 1). Height Z-score and eGFR at start of RRT of PBS patients were similar to controls (Table 2).

**Table 2:** Clinical and demographic characteristics of patients at the start of renal replacement therapy

	Prune belly syndrome (*n* = 88)	Congenital obstructive uropathy (*n* = 904)	Congenital renal hypo−/dysplasia (*n* = 1128)	*P* value*
Height (z-scores)	-1.69 (−3.29 to −0.67) *n* = 48	−1.88 (−2.76 to −0.94) *n* = 485	−1.88 (−2.85 to −1.04) *n* = 516	0.65^a^
eGFR	8.61 (6.61–11.00) *n* = 35	8.18 (6.39–10.30) *n* = 354	8.18 (5.97– 10.64) *n* = 437	0.78^a^
Age	7.0 (0.9–12.2)	9.6 (3.0–14.1)	9.4 (2.7– 14.2)	0.02^a^
Age groups* n* (%)				
< 0.5 years	15 (17.1%)	77 (8.5%)	120 (10.6%)	0.04^b^
0.5–5 years	25 (28.4%)	253 (28.0%)	288 (25.5%)	
6–10 years	22 (25.0%)	183 (20.2%)	250 (22.2%)	
≥11 years	26 (29.5%)	391 (43.3%)	470 (41.7%)	
RRT periods* n* (%)^1^				
1990–1999	32 (49.2%)	312 (48.6%)	298 (39.3%)	0.002^b^
2000–2013	33 (50.8%)	330 (51.4%)	460 (60.7%)	
Initial RRT modality* n* (%)				
HD	15 (17.1%)	236 (26.1%)	287 (25.4%)	0.21^b^
PD	44 (50.0%)	377 (41.7%)	452 (40.1%)	
Tx	29 (33.0%)	277 (30.6%)	363 (32.2%)	
Missing	0 (0%)	14 (1.6%)	26 (2.3%)	

Medians (interquartile range) are given for continuous variables; frequencies and percentages are given for categorical variables

*eGFR* estimated glomerular filtration rate,* RRT* renal replacement therapy,* HD* hemodyalisis,* PD* peritoneal dialysis,* Tx* transplantation

^1^Among countries with complete coverage over the entire follow-up period (Austria, Croatia, Denmark, Finland, Greece, Iceland, Norway, Spain, Sweden, Switzerland, The Netherlands, United Kingdom)

*P values are based on Kruskal-Wallis test^a^ and chi-square test^b^

**Fig. 2:**
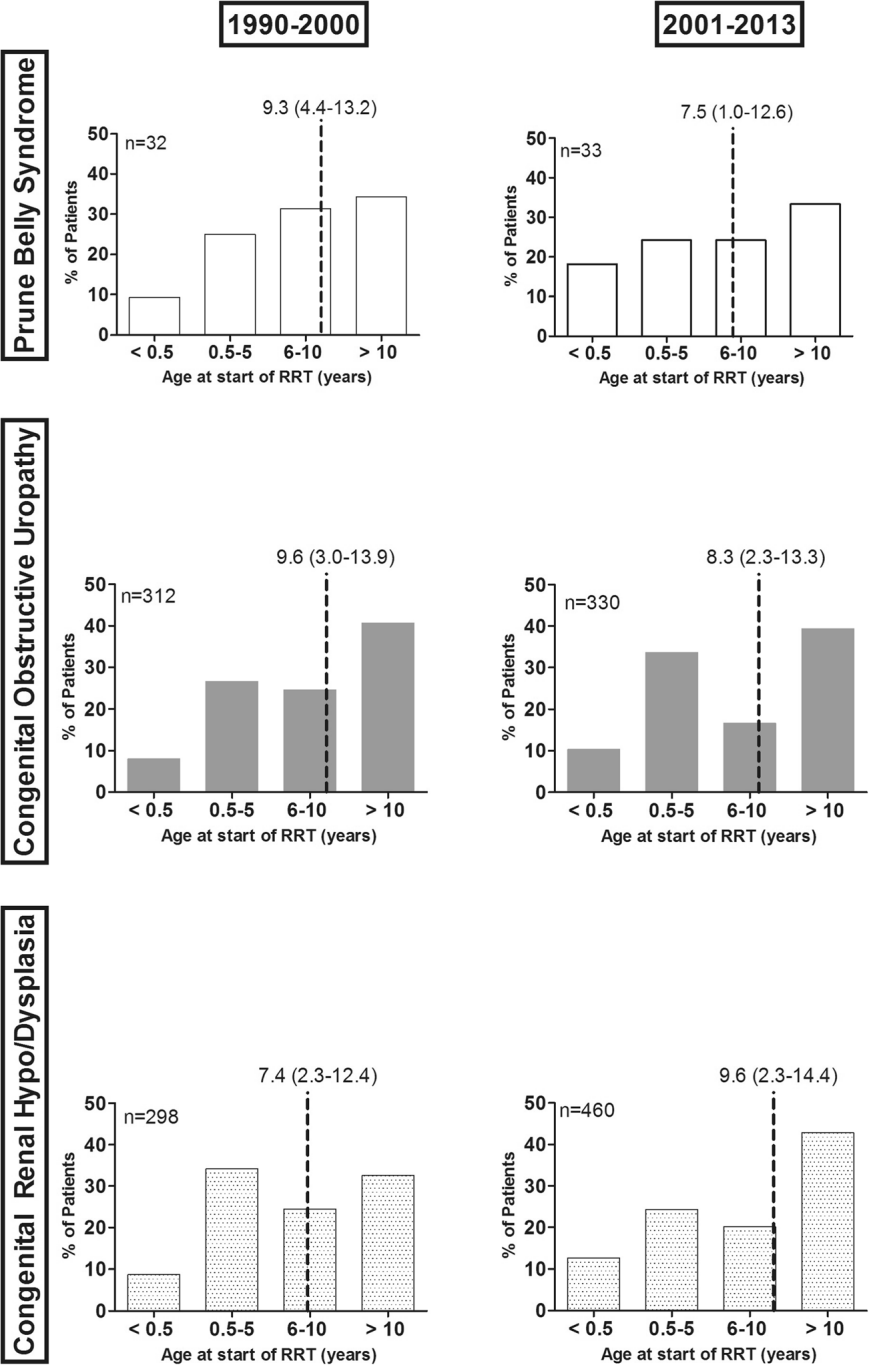
Age at renal replacement therapy (RRT) onset over the two time periods. Only countries with complete follow-up are included in the analyses.* Dotted lines* indicate median age [interquartile range (IQR)] at start of RRT

Among countries with complete follow-up (*n* = 1465), comparison between the 1990–2000 and the 2001 and 2013 cohort, there was a trend towards starting RRT at younger ages among PBS and COU patients but not in RHD patients (Fig. 2). In the RHD group, there was a shift over time to older ages at RRT onset.

The most frequent treatment modality when commencing RRT for PBS was PD (50%), followed by renal transplantation (33%) and HD (17%) (Table 2). Distribution of treatment modalities when commencing RRT was similar in PBS when compared with controls.

### Patient survival

During the observation period, 111 patients died, nine of whom had PBS. Causes of death of PBS patients were similar to those of controls. Infection was the most frequent known cause of death (44% PBS, 42% COU, 20% RHD) (Table 3).

**Table 3:** Number and causes of deaths in the study population

	Prune belly syndrome (*n* = 88)	Congenital obstructive uropathy (*n* = 904)	Congenital renal hypo/dysplasia (*n* = 1128)
Death	9 (10.2%)	36 (4.0%)	66 (5.9%)
Cause of Death			
Fluid overload/pulmonary edema	0 (0%)	0 (0%)	3 (4.5%)
Hemorrhage	0 (0%)	1 (2.8%)	1 (1.5%)
Cardiac arrest	1 (11.1%)	3 (8.3%)	6 (9.1%)
Cerebrovascular accident	0 (0%)	0 (0.0%)	3 (4.5%)
Infections	4 (44.4%)	15 (41.7%)	13 (19.7%)
Malignancies	0 (0%)	0 (0%)	5 (7.6%)
Uremia caused by graft failure	0 (0%)	0 (0%)	4 (6.1%)
Other known	2 (22.2%)	11 (30.6%)	15 (22.7%)
Treatment withdrawal	2 (22.2%)	2 (5.6%)	3 (4.5%)
Unknown/missing	0 (0.0%)	4 (11.1%)	13 (19.7%)

**Fig. 3:**
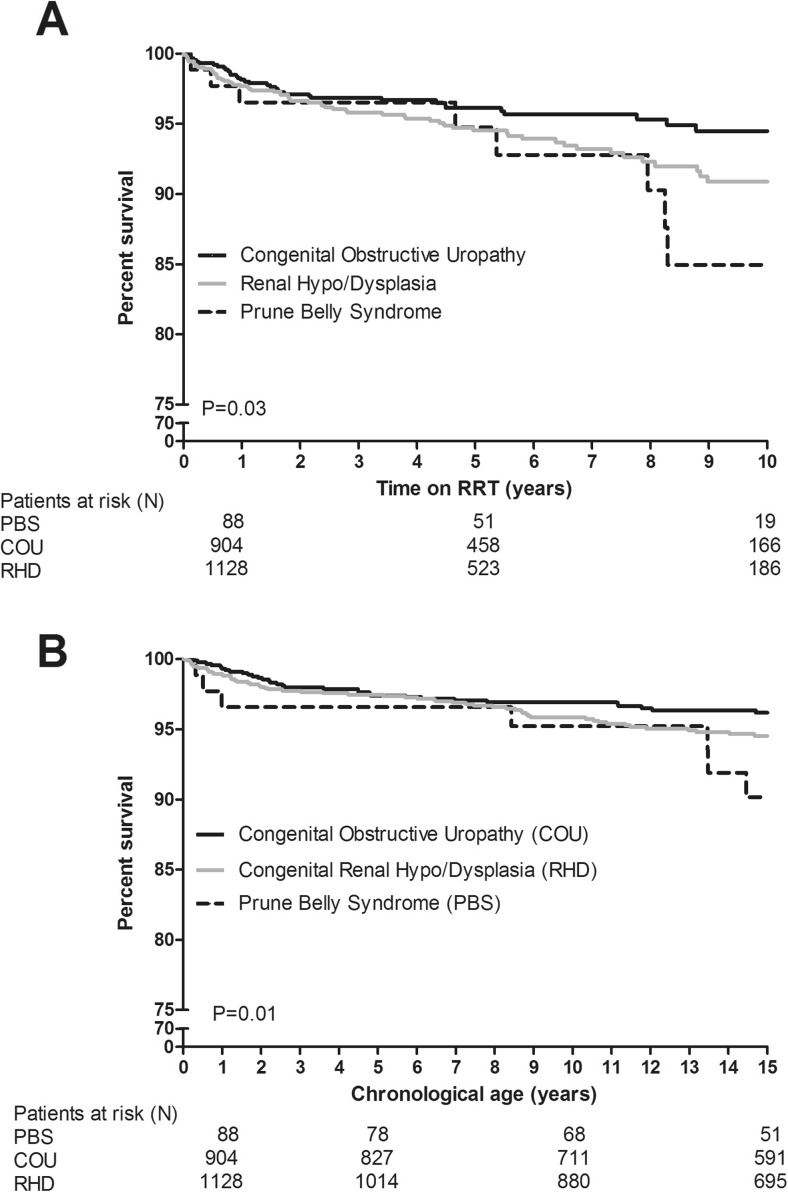
Unadjusted survival of patients with prune belly syndrome (PBS) and control groups according to time on renal replacement therapy (RRT) (**a**) and according to chronological age (**b**)

Among PBS patients, the overall 1-year survival was 86.7%, 100%, and 100% at ≤1 year, 1–12 years, and >12 years at start of RRT, respectively; 5-year survival rates were 86.7%, 100%, and 80.0%, respectively; overall 10-year survival rates were 85% for PBS, 94% for COU, and 91% for RHD. Unadjusted survival since start of RRT (Fig. 3A) and according to chronological age (Fig. 3b) differed significantly between groups.

Mortality risk of PBS patients was significantly higher than those with COU [hazard ratio (HR) 2.27; 95% confidence interval (CI) 1.30–3.98] but similar to patients with congenital RHD (HR 1.48; 95% CI 0.70–3.14). Adjusting for country, age, and period of RRT did not change these associations [adjusted HR (aHR) PBS vs. COU 2.21; 95% CI 1.03–4.77; aHR PBS vs. RHD 1.46; 95% CI 0.70–3.07].

### PD technique survival

Of the 873 patients who started RRT on PD, 103 (eight PBS, 44 COU, 51 RHD) experienced technique failure or switched to HD after a median of 1.2 (IQR 0.7–2.4) years on PD. One-year technique survival was 92.1%, 93.1%, and 93.7% for PBS, COU, and RHD patients, respectively. Age, country, and period-adjusted risk of PD failure was not significantly different between groups (aHR PBS vs. COU 1.17, 95% CI 0.50–2.72; aHR RHD vs. COU 0.97, 95% CI 0.76–1.24).

### Transplantation

During the follow-up period, 74 (84.1%) PBS patients received a first kidney transplant at a median age of 9.3 years (IQR 4.7–13.2). The median time on dialysis before first kidney transplantation was 8.4 months (IQR 0.0–21.1), whereas 29 (33.0%) patients received a pre-emptive kidney graft. Time spent on dialysis before transplantation was similar in PBS and control groups (Table 4), as was the chance of receiving a first kidney transplant (aHR 1.00; 95% CI 0.78–1.29).

**Table 4:** Characteristics of patients receiving their first kidney transplant (Tx)

	Prune belly syndrome	Congenital obstructive uropathy	Congenital renal hypo−/dysplasia	*P* value
N receiving a Tx	74 (84.1%)	734 (81.2%)	892 (79.1%)	0.17^b^
Age at Tx (years)	9.3 (4.7–13.2)	10.1 (4.7–14.1)	9.8 (4.9–14.6)	0.48^a^
Time to Tx (months)	8.4 (0.0–21.1)	5.7 (0.0–17.3)	4.8 (0.0–16.1)	0.28^a^
Known Donor Type				
Deceased	45 (60.8%)	458 (62.4%)	520 (58.3%)	0.21^b^
Living	22 (29.7%)	258 (35.2%)	344 (38.6%)	

Medians (IQR) are given for continuous variables, frequencies and percentages are given for categorical variables;

^a^Kruskal–Wallis test, ^b^chi-square test

Most patients received a kidney from a deceased donor, with little difference between PBS and controls (60.8%, 62.4%, and 58.3% in PBS, COU, and RHD, respectively). Five PBS patients (6.8%) and 153 (9.4%) controls received two or more transplants during follow-up. Competing risk analysis demonstrated that PBS patients had a similar likelihood of receiving their first kidney transplant within 2 years after commencing RRT (65%) as controls: 71% in COU and 68% in RHD. Within 5 years, this was 87% in PBS, 88% in COU, and 85% in RHD. Unadjusted 5- and 10-year graft survival was 86.2% and 78.4% in PBS, 86.5% and 75.9% in COU, and 87.0% and 74.5% in RHD, respectively. After adjustment for country, age at and period of transplantation, death-censored risk of graft loss was not significantly different between PBS and COU patients (aHR 0.94, 95% CI 0.53–1.66) or RHD (aHR 0.91, 95% CI 0.51–1.62). Similar results were obtained when death was included as graft failure event.

## Discussion

In this study, we provide an analysis of the largest reported cohort to date of PBS patients— 88 boys <20 years when commencing RRT—and showed that outcomes were better than previously reported. PD technique and graft survival rates were similar to boys on RRT due to other forms of CAKUT. Patient survival of male PBS patients was similar to that of male RHD patients but significantly lower than males on RRT due to COU.

Median age at start of RRT was significantly lower in boys with PBS (7.0 years) compared with those with COU (9.6 years) and RHD (9.4 years). The relatively high incidence in patients <6 months can possibly be attributed to significant renal dysplasia, while the later peak, between 6 and 10 years, is hypothesized to occur due to additional renal damage caused by repeated infections or high pressures generated from obstruction. This clinical information has not been reported to the Registry. Nearly 45% of patients with COU started dialysis after 10 years of age, whereas >40% of PBS patients needed dialysis before 5 years of age, including almost 20% who started within the first 6 months of life, suggesting that severity of dysplasia rather than obstruction plays the most important role in the pathogenesis and predominantly determines the renal outcome of PBS patients.

In the past 25 years, there was a trend over time toward a decreased age at start of RRT in patients with PBS. The higher proportion of very young patients in the last decade may reflect the increasing acceptance of infants into RRT programs and increased awareness of the disease. The overall 85% 10-year patient survival rate in PBS patients on RRT was rather good and similar to those with RHD but slightly worse compared with COU. However, it should be noted that PBS patients entering RRT programs probably are the ones with more favorable prognosis. Conversely, PBS is associated with severe comorbidities that may affect survival on RRT; only limited information on comorbidities is available from the Registry. Causes of death in our study were similar in all groups, with infection being the most frequent cause; fewer patients died of cardiovascular causes. As the presence of severe renal dysfunction and pulmonary hypoplasia results in mortality in nearly all PBS patients within a short time, infants who survive frequently have adequate urine production, which might protect them from fluid overload, hypertension, and other cardiovascular problems [17, 18]. Nevertheless, our study clearly demonstrates that survival of PBS patients on RRT is encouraging, and RRT should be offered to PBS patients, including infants.

We encountered no restrictions to performing PD in boys with PBS due to hypoplasia of the abdominal wall musculature. PD frequency as the initial treatment modality and PD technique survival were similar for PBS, COU, and RHD patients.

Renal transplantation appears as the best treatment option for PBS patients with ESRD [18, 19]. A few single-center studies have assessed the outcome of renal transplantation in children with PBS; most were retrospective, deceased-donor transplant reports on a small number of cases ranging from five to nine patients [9, 20, 21]. Our study involved 74 children and 81 grafts (30% living donors) with a median follow-up of >5 years. Accordingly, this is the first large, population-based study providing comprehensive information on timing and outcomes of transplantation in PBS. Median age at transplantation was 9.3 years—comparable with previous reports [9, 20, 22]. Time spent on dialysis before transplantation was similar in PBS and control groups, as was the chance of receiving a first renal transplant. In addition, 65% of PBS patients had their first kidney transplant within 2 and 87% within 5 years after commencing dialysis; one third received a pre-emptive kidney graft. Reinberg et al. [21] found that patients with PBS waited a shorter period for transplantation than controls and suggested that the distensible abdominal wall characteristic of the syndrome permits placement of an adult kidney in a young child. Previous reports have compared the outcome of transplanted PBS patients with the outcome of age-matched controls, including or excluding patients with a dysfunctional lower urinary tract or a nondiabetic cause of ESRD [20–22]. Five-year graft survival rates reported by Fontaine et al. [22] [22], Fusaro et al. [9], and Kamel et al. [20] were 50%, 67%, and 73%, respectively, and were similar to control groups. We found remarkably better graft survival in our cohort, with 5- and 10-year survival rates in PBS of 86% and 78%, respectively.

To the best of our knowledge, we provide the first comprehensive information on outcomes of the largest population-based cohort of PBS patients requiring RRT to date. However, some limitations need to be acknowledged. Due to the nature of the Registry, including only children on RRT, our data underestimate the true incidence of PBS in Europe and results only apply to male PBS patients on RRT. The severity of urinary tract abnormalities in PBS patients and rate of urinary tract infections and surgical management are not reported to the Registry and could therefore not be studied. Furthermore, detailed information on comorbidities was not available. In addition, a major fraction of patients in the COU group most likely had posterior urethral valves (PUV), as it is the most common obstructive uropathy in boys leading to ESRD. However, because of the lack of detail in primary renal disease coding, we cannot fully ensure that all COU patients had PUV.

## Conclusions

In conclusion, in this Europe-wide study of boys with PBS on RRT, we demonstrate that outcomes are similar to those of patients on RRT due to other forms of CAKUT. Graft and overall survival of PBS patients are encouraging, suggesting that RRT should be offered to all PBS patients similarly to that offered to patients with other forms of CAKUT.
